# Primary architecture and energy requirements of Type III and Type IV secretion systems

**DOI:** 10.3389/fcimb.2023.1255852

**Published:** 2023-11-27

**Authors:** Elena Cabezón, Fernando Valenzuela-Gómez, Ignacio Arechaga

**Affiliations:** Departamento de Biología Molecular and Instituto de Biomedicina y Biotecnología de Cantabria (IBBTEC), Universidad de Cantabria- CSIC, Santander, Spain

**Keywords:** T3SS, T4SS, bacterial conjugation, effector delivery, ATPases

## Abstract

Many pathogens use Type III and Type IV protein secretion systems to secrete virulence factors from the bacterial cytosol into host cells. These systems operate through a one-step mechanism. The secreted substrates (protein or nucleo-protein complexes in the case of Type IV conjugative systems) are guided to the base of the secretion channel, where they are directly delivered into the host cell in an ATP-dependent unfolded state. Despite the numerous disparities between these secretion systems, here we have focused on the structural and functional similarities between both systems. In particular, on the structural similarity shared by one of the main ATPases (EscN and VirD4 in Type III and Type IV secretion systems, respectively). Interestingly, these ATPases also exhibit a structural resemblance to F_1_-ATPases, which suggests a common mechanism for substrate secretion. The correlation between structure and function of essential components in both systems can provide significant insights into the molecular mechanisms involved. This approach is of great interest in the pursuit of identifying inhibitors that can effectively target these systems.

## Introduction

Many Gram-negative pathogens encode virulence-associated Type III and Type IV secretion systems (T3SS and T4SS, respectively), which they use to translocate effector molecules. These systems are highly optimized multi-protein machineries that translocate effectors from the bacteria directly into the host cell cytoplasm, enabling effective manipulation of host processes (Galán and Waksman, 2018; Viana et al., 2021). T3SSs can be easily found in pathogenic bacteria such as *Escherichia coli*, *Yersinia*, *Salmonella*, *Chlamydia* or *Pseudomonas* (Cornelis, 2006; Coburn et al., 2007; Cornelis, 2010). T4SSs are associated to pathogens like *Helicobacter*, *Legionella*, *Neisseria* or *Coxiella*, among others (Cascales and Christie, 2003). Plant pathogens, such as *Pseudomonas* or *Agrobacterium* can also have T3- and T4SSs, respectively.

Apart from being involved in virulence, these secretion systems have other associated functions. For instance, flagellar systems, which have dynamic flagella to drive cellular motility, are related to T3SSs. In this case, the substrates translocated across the cell membranes are the components of the flagellum during biogenesis. The origin, specialization and diversification of these two different T3SS remain elusive, but they both share a high degree of homology, suggesting a common evolution from machines involved in motility (Gophna et al., 2003; Saier, 2004; Pallen et al., 2005; Abby and Rocha, 2012; Hu et al., 2017). As it happens in T3SSs, T4SSs are also versatile nanomachines, which are not only involved in virulence but also in DNA and nucleo-protein substrates delivery into bacterial or eukaryotic target cells, via a contact-dependent mechanism (Christie et al., 2014; Cabezón et al., 2015). They are ancestrally related to DNA conjugation machines involved in horizontal gene transfer and virulence (Guglielmini et al., 2013). In both cases, the architecture of these secretion systems is relatively well preserved regardless of its biological function.

T3 and T4SSs have several important features in common. They both span the inner membrane (IM), periplasm, and outer bacterial membrane (OM). Thus, the mechanism of transport involves just one step, which means that substrates are directly delivered into the host cell without the assistance of the Sec or Tat machineries (hence known as Sec- or Tat-independent protein secretion) (Christie, 2019). The internal diameter of the channel is similar in both secretion systems, less than 30 Å, which means that substrates must be unfolded to be secreted. Accordingly, both systems have specialized ATPases at the base of the secretion channel, which provide energy for substrate unfolding (T3SS) or pilus biogenesis and substrate transport (T4SS). Yet, many of the underlying molecular mechanisms that power secretion remain unclear.

The aim of this review is to provide a general overview of the two systems. Analyzing the structure-function relationship and comparing key elements shared by both systems can be extremely valuable in understanding the molecular mechanism and, more importantly, in the search of inhibitors that could target these systems.

## Overview of a prototypical T3SS architecture

In recent years, cryo-electron microscopy advances have allowed the determination of the architecture of these two secretion systems revealing large, megadalton-sized macromolecular assemblies (Hu et al., 2018; Lunelli et al., 2020; Macé et al., 2022).

**Figure 1:**
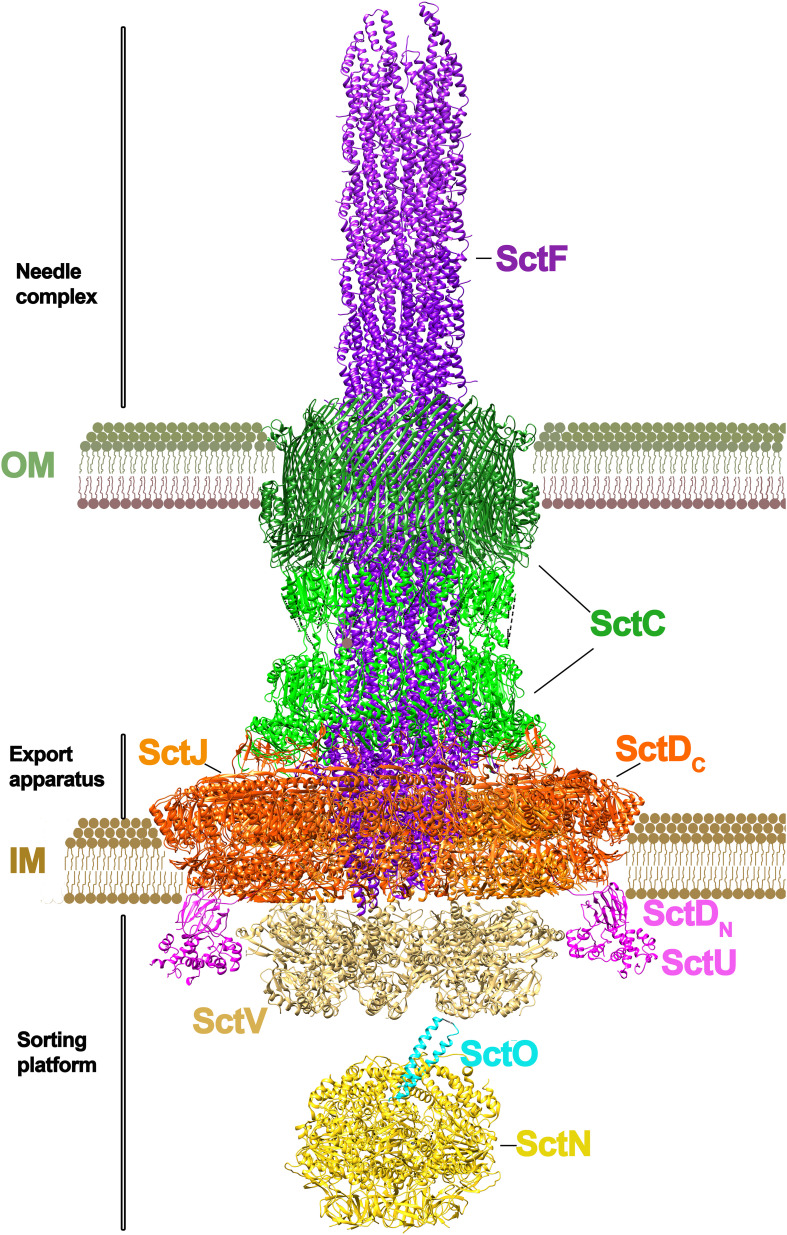
Architecture of a T3SS. The T3SS of *Salmonella* sp. is shown. The macromolecular assembly spans the inner (IM) and outer membranes (OM). Two distinct parts can be differentiated in the system: the needle complex and the sorting platform (Lara-Tejero and Galán, 2019; Soto et al., 2022). At the base of the needle complex there is a multi-ring structure that spans the inner membrane, formed by PrgH and PrgK subunits (SctD and SctJ subunits in the unified nomenclature). The IM base is connected to the outer membrane by an inner rod protein (PrgJ/SctI). At the outer membrane there is the secretin ring formed by InvG (SctC), where the needle filament protein (PgrI/SctF) is attached. The length of the ring is regulated by InvJ (SctP). The export apparatus (no shown) is formed by a number of small proteins (SpaP/SpaQ/SpaR in *Salmonella*; SctR/SctS/SctT in the unified nomenclature) connected to the C-ring (SpaO/SctQ) at the base of the channel by an export apparatus switch protein (SpaS/SctU). The export apparatus sorting complex is formed by a nonameric protein ring (InvA/SctV). The unfolding of the substrates is powered by a hexameric ATPase (InvC/SctN). In the lumen of this ATPase sits a stalk protein (InvI/SctO). (The figure has been created by using the PDB codes: 7ah9.pdb (Miletic et al., 2021), 7awa.pdb (Matthews-Palmer et al., 2021), 7k08.pdb (Majewski et al., 2019), 6uid.pdb and 6uie.pdb (Muthuramalingam et al., 2020).

T3SSs, also known as injectiosomes for their syringe-shape, are large multiprotein complexes that span the inner and outer membrane of bacteria (**Figure 1**). Much of the structural knowledge about this secretion system is available from *Salmonella* and, therefore, we will use its nomenclature in this review accompanied of the unified nomenclature in parenthesis. A complex formed by eight membrane proteins spans the bacterial inner membrane (IM), periplasm and outer membrane (OM). The outer membrane protein InvG (SctC) belongs to the secretin family of β–barrel pores and it forms a ring with a 15-fold symmetry (Hu et al., 2018). In contrast, the IM ring, formed by SpaPQRS (SctRSTU) and PrgHK (SctDJ) proteins presents a 24-fold stoichiometry. Recent cryo-EM structure of the complex shed light on this symmetry mismatch puzzle between the OM and IM rings, revealing that the InvG ring incorporates an additional InvG monomer at the periplasmic site (Hu et al., 2019). This local variation in stoichiometry facilitates the interaction with the IM components.

Associated to this macromolecular complex is InvA (StcV), a transmembrane protein with a large globular domain located in the cytosol. The crystal structure of this globular domain (MxiA in *Shigella fastidiosa*) (Abrusci et al., 2013), as well as more recent tomography (Hu et al., 2017) and cryo-EM studies (Majewski et al., 2019; Kuhlen et al., 2021) confirm that this protein assembles as a nonameric complex. This InvA(SctV) protein, together with other small proteins such as OrgA(SctK), OrgB(SctL), SpaO(SctQ), InvI(SctO) and the ATPase InvC(SctN) form the sorting platform. The role of this platform, linked to the export apparatus, is to select effector proteins and target them for secretion. The hexameric ATPase, InvC (SctN) is involved in the unfolding of the effector proteins prior secretion (Akeda and Galán, 2005; Bergeron and Marlovits, 2022). The export apparatus SpaPQRS forms a right-handed pseudo-helical assembly, which works as a platform for PrgIJ (SctFI) needle polymerization. PrgJ (SctI) contacts with SpaPR and secretin InvG are required for needle assembly, thus providing the platform for a further polymerization of the PrgI (SctF) needle (**Figure 1**).

## Overview of a prototypical T4SS architecture

**Figure 2:**
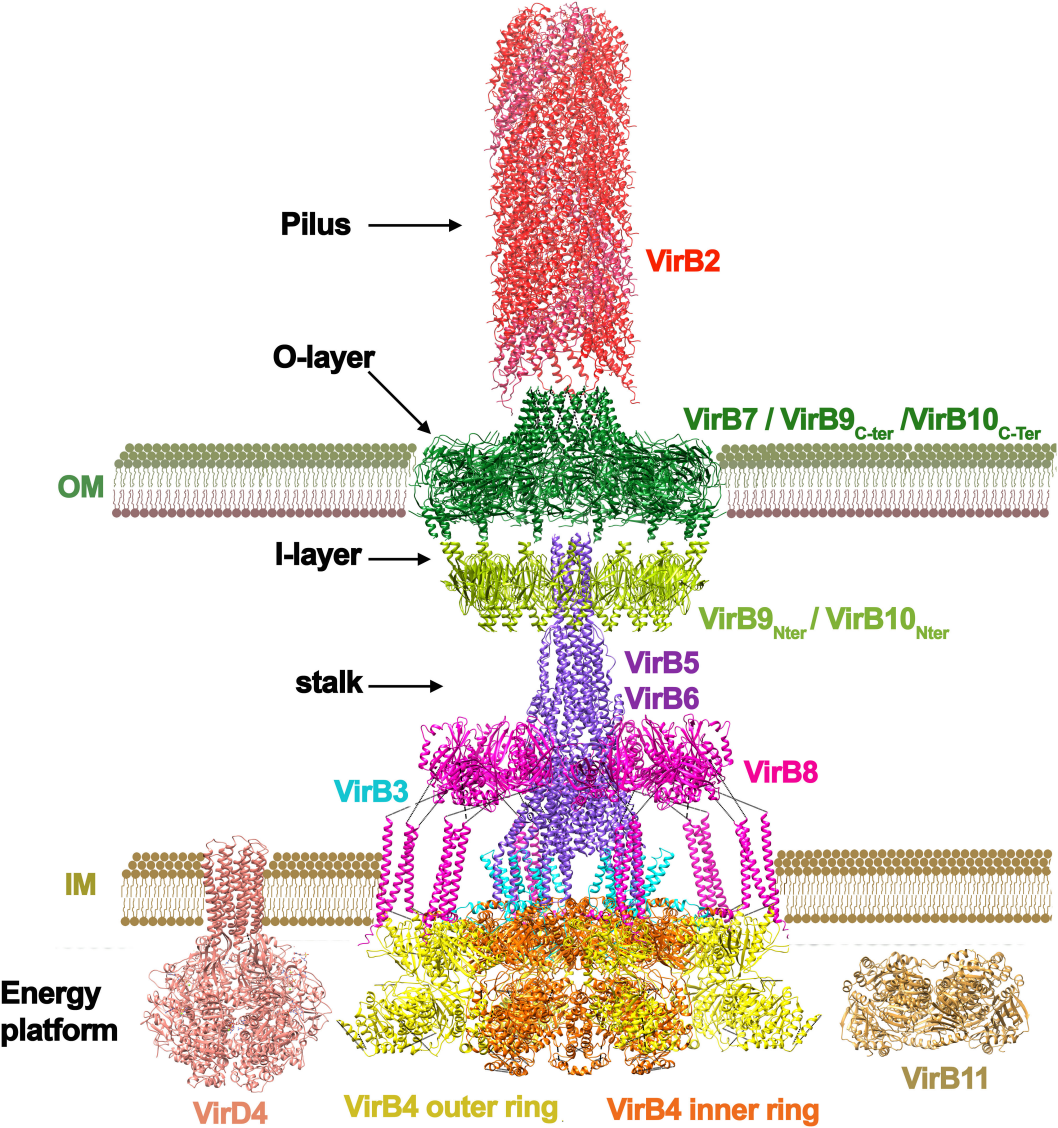
Architecture of a T4SS. The T4SS of the conjugative plasmid R388 is shown (Macé et al., 2022) from PDB codes: 7o3.pdb, 7o3j.pdb, 7o3v.pdb, 7q1v.pdb, 7o43.pdb, and 7oiu.pdb. The subunits are named following the *Agrobacterium tumefaciens* nomenclature. The system is formed by 11 different proteins (named VirB1 to VirB11). The main ATPase of the system is VirB4 (TrwK in R388), which forms a hexamer of dimers at the base of the channel. This ATPase is thought to power pilus formation but also substrate secretion. Conjugative systems have an additional ATPase, named coupling protein (Cabezon and de la Cruz, 2006), which is not present in the cryo-EM structure (pdb 1e9r.pdb taken from (Gomis-Rüth et al., 2001). Most T4SS also have another ATPase named VirB11 (TrwD in R388) suggested to act in the unfolding of the substrate (pdb obtained by molecular modelling as shown in (Ripoll-Rozada et al., 2013). VirB4 (TrwK) is attached to the inner membrane by VirB3 (TrwM), an integral membrane protein. The inner membrane platform also contains the N-terminal domain of VirB8 (TrwG), a protein that spans towards the periplasmic space forming an arched shaped structure. The periplasmic space protein (VirB6) forms a stalk structure that connects the inner membrane complex with the outer membrane ring formed by VirB7 (TrwH in R388), VirB9 (TrwF) and VirB10 (TrwE). Conjugative systems contain a pilus attached to the outer membrane formed by an oligomeric helical structure of pilin subunits (VirB2/TrwL). This pilus structure has variable length depending on the system. It is important to note that the pilus structure shown in red corresponds to that of F plasmid (pdb 5leg.pdb from (Costa et al., 2016), solved independently of the secretion system. As mentioned in the main text, the solved T4SS structure shown here might correspond to a state involved in pilus biogenesis (Macé et al., 2022), which would explain why VirB5 appears in the periplasmic space being a specific adhesin protein expected to be localized at the tip of the pilus (Aly and Baron, 2007).

T4SSs are also a heterogeneous group of nanomachines and can be classified in two main groups: conjugation systems and effector translocation systems (Sheedlo et al., 2022). Most simple T4SS consist of 12 proteins (named VirB1-VirB11 and VirD4 in the *Agrobacterium tumefaciens* unified nomenclature), also known as “minimized T4SSs” (Sheedlo et al., 2022) (**Figure 2**). Most recent T4SS structural data comes from the conjugative plasmid R388 (Macé et al., 2022), so this nomenclature will be used in this review, accompanied from the unified nomenclature in parenthesis. As it occurs in T3SSs, here it is also evident the existence of symmetry mismatches in the core complex. Whereas in T3SSs the OM ring is formed only by the secretin protein, three proteins are involved in the OM complex in T4SSs: TrwH (VirB7), TrwF (VirB9) and TrwE (VirB10), forming a ring with a barrel-shape architecture and a 14-fold symmetry. TrwH is completely embedded in the OM, but the N-terminal domains of TrwE and TrwF proteins also expand into the periplasmic space forming what is called the “I-layer”, differentiated from the embedded part in the OM, called “O-layer”. Intriguingly, the periplasmic N-terminal domains of these two proteins (the I-layer) form a 16-fold complex, which means that the C-terminal domains of these two heterodimers are not inserted in the O-layer. Similar symmetry mismatches have also been reported in protein translocating T4SSs, such as the C17:C14 mismatch found in *H. pylori* (Chung et al., 2019; Sheedlo et al., 2020) or a C18:C13 mismatch in *L. pneumophila* (Durie et al., 2020).

In T3SSs, this local variation in stoichiometry is thought to facilitate the interaction with the IM components. However, in T4SSs, the OMC complex does not contact directly with the IM complex. Instead, a structure called “stalk” bridges the core of the OM and the IM complexes. This central, cone-shaped structure is composed of a pentamer of VirB6 (TrwI) inserted into the inner membrane, and a pentamer of VirB5 (TrwJ) mounted onto the VirB6 stalk base (Macé et al., 2022). The location of VirB5(TrwJ) has also been described to be at the T-pilus tip in the *A. tumefaciens* T4SS (Aly and Baron, 2007), which suggests that VirB5, also known as adhesin, changes position depending on the stage of the conjugation process. It is important to note that in the T4SS structure solved by cryo-EM (Macé et al., 2022) the pilus is absent. The pilus structure shown in **Figure 2** corresponds to that of F plasmid (Costa et al., 2016), solved independently of the secretion system. In addition, it is also worth noting that in some T4SS, such as the Dot/IcM system from *Legionella*, an extended pilus has never been observed. Last findings suggest that, in this system, effector translocation occurs by close membrane contact (Böck et al., 2021).

In the cryo-EM structure, the stalk is surrounded by a ring complex, known as the arches, which is composed of hexamers of homotrimeric units of the periplasmic domain of VirB8 (TrwG). VirB8 protein contacts one of the main components of the IM complex: the VirB4 ATPase (TrwK). VirB4 forms a hexamer of dimers. One of the subunits of each dimer is involved in the formation of a central hexamer, attached to the inner membrane through VirB3 (TrwM). The second subunit of the dimers protrudes out, and it makes contacts with the tails of three VirB8 subunits. This oligomeric arrangement has only been found in VirB4 ATPases and it is still a controversial issue, since a previous work by electron microscopy showed this protein organized into 2 side-by-side hexameric barrels (Low et al., 2014).

It is worth noting that the described structure corresponds to a simple “minimized T4SS” (Costa et al., 2021). Examples of “expanded T4SSs” include the *H*. *pylori* Cag T4SS (Cover et al., 2020), the *L*. *pneumophila* Dot/Icm (Chetrit et al., 2018; Park et al., 2020) T4SS, and the F plasmid-encoded T4SS (Liu et al., 2022). Expanded systems have a larger size and require more proteins. In addition, none of the T4SS structures solved so far reveals a continuous central channel. OM and IM complexes are only connected by the projecting pilus structure and, therefore, the solved structures might correspond to a state involved in pilus biogenesis (Macé et al., 2022), but not in DNA-protein transfer; a state in which VirD4 (TrwB) ATPase would also be required. In addition, TrwD, a traffic ATPase related to the T2SS EpsE/PilB/PilT proteins is also missing in this cryo-EM structure.

## Energy supply for substrate transport

Translocation of proteins and protein-DNA complexes across membranes is a process that requires energy. T3SS and T4SS rely on several associated ATPases and/or the proton motive force (PMF) to drive translocation (Christie, 2019). Nonetheless, the details of the secretion mechanism are still unclear.

The limited diameter of the T3SS needle channel (~ 2 nm) (Lyons et al., 2021) reflects that protein effector unfolding must occur for substrate translocation, unless the channel opens dramatically during translocation, which would compromise cell viability. In this sense, many T3SS effectors interact with chaperones, which maintain these substrates in an unfolded and secretion-competent state prior to translocation (Stebbins and Galán, 2001; Lee and Galán, 2004; Akeda and Galán, 2005). The T3SS hexameric ATPase InvC (SctN), at the base of the channel, would mediate T3SS effectors unfolding before secretion (Deng et al., 2017). It is thought that ATP hydrolysis is required for the initial step of substrate secretion, but additional force is provided by the PMF (Minamino et al., 2008) via the proton channel SctV (Minamino et al., 2011). Therefore, T3SS depends on ATP and PMF energy sources for efficient secretion (Wilharm et al., 2004; Erhardt et al., 2014; Halte and Erhardt, 2021). Nevertheless, these issues are still highly controversial. There is a large distance (over 30 nm) between the ATPases within the cytosolic side and the cell wall (Matias et al., 2003) and this primary energy supply would be inefficient for substrate transfer (Grange et al., 2008). In that sense, folding of the secreted substrate upon exit from the secretion channel might also provide energy for pulling protein effectors through the apparatus (Lee and Rietsch, 2015).

**Figure 3:**
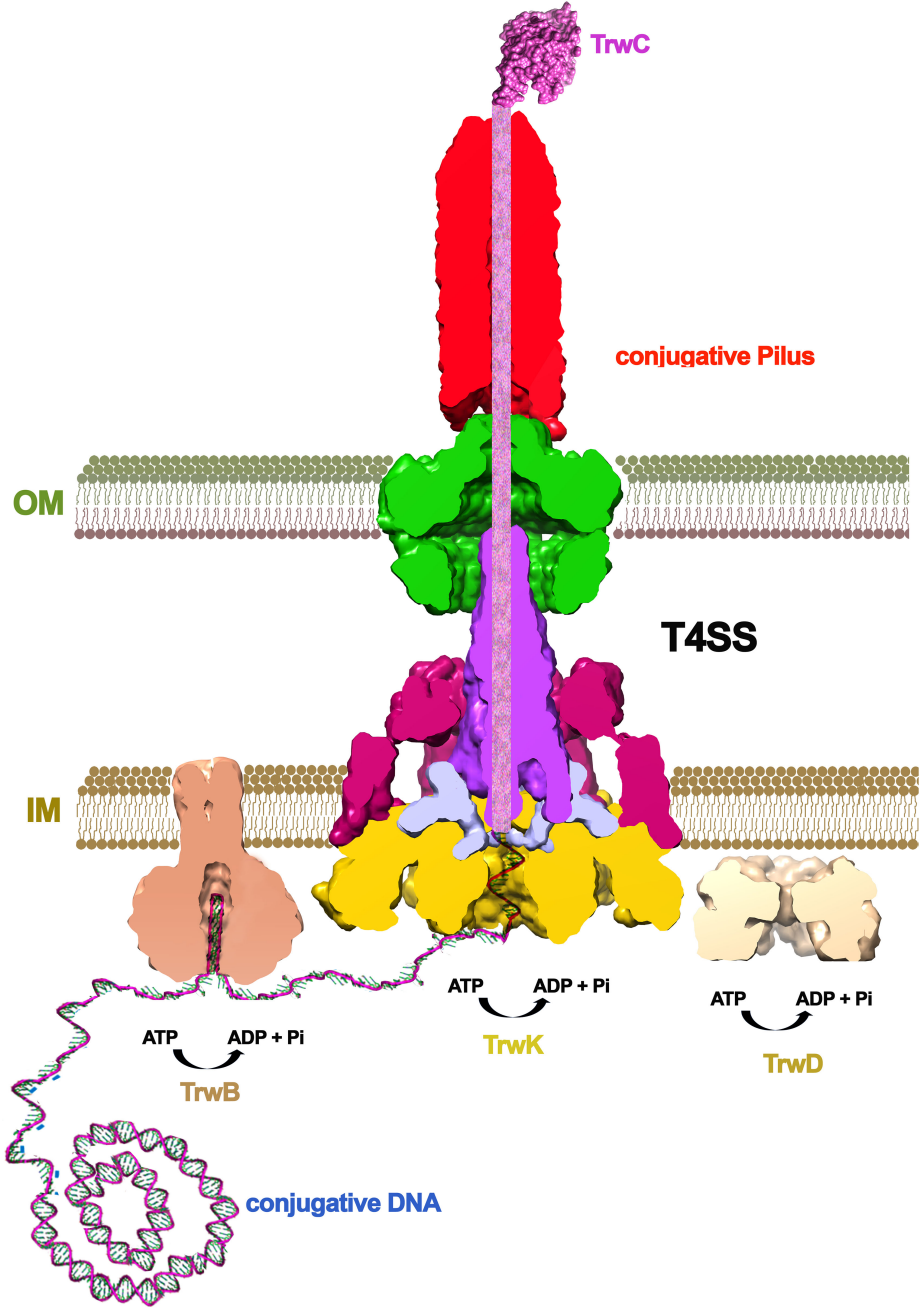
The conjugative mechanism. Bacterial conjugation is triggered by a signal (probably the contact with a recipient cell), which induces the binding of the relaxase protein (TrwC in R388) to the conjugative plasmid. After nicking the DNA, the relaxase remains covalently bound to the DNA, which is unwinded by the helicase domain within the same protein. DNA processing is helped by the coupling protein (VirD4/TrwB), which presents the nucleo-protein substrate to the base of the channel. TrwC relaxase is a huge protein of 966 amino acid residues (1,756 in the case of F plasmid) that has to be unfolded to cross the inner and outer membranes of both, the donor and the recipient cell. This unfolding is thought to be powered by VirB11 (TrwD), an ATPase related to T2SS proteins PilB/PilT/GspE.

**Figure 4:**
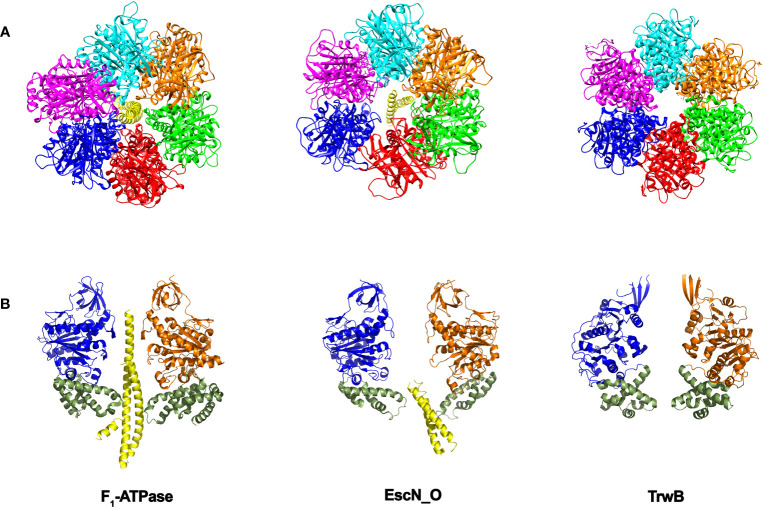
Structural similarities between secretion ATPases and F1-ATPase. As the structures of the secretion ATPases were unveiled, a striking similarity with the F_1_-ATPase structure (Abrahams et al., 1994) was emerging. The structure of the enteropathogenic *E. coli* T3SS ATPase EscN (Majewski et al., 2019), revealed an hexameric ring with a central stalk EscO that resembles the structure of the α/β ring with its central γ stalk in F_1_-ATPase. The structure of the T4SS coupling protein TrwB (Gomis-Rüth et al., 2001) also resembles F_1_-ATPase (Cabezon and de la Cruz, 2006). **(A)** Bottom view. Each subunit in the ring is shown in a different color. **(B)** Side view. For more clarity, only two opposite subunits of the ring are shown. The “all-α-domain” in TrwB is shown in green (residues 184–297). The equivalent domain in F_1_-ATPase corresponds to residues 364–474, which constitute a mobile domain that acquires a different conformation in the three catalytic subunits and contains the residues involved in the interaction with the γ-subunit. The equivalent domain in EscN ATPase includes residues 371-446. TrwB lacks a central coiled-coil subunit. Instead, one single DNA strand might be translocated through the internal cavity. DNA translocation could be driven by the movement of the “all-α-domain” as a consequence of ATPase activity (by analogy to F_1_-ATPase). (The figure has been created by using the PDB codes: 1e1q.pdb and 1e1r.pdb (Braig et al., 2000), 6njp.pdb (Majewski et al., 2019), and 1e9r.pdb (Gomis-Rüth et al., 2001).

T3SS likewise, the T4SS structure also reveals a central channel with a narrow internal diameter (~2 nm)(Macé et al., 2022). Thus, substrates must be unfolded in order to be transported. Three ATPases power T4SS mediated secretion: VirD4 (TrwB), VirB4 (TrwK) and VirB11 (TrwD). All three are essential for bacterial conjugation in the reference plasmid R388 (**Figure 3**). Of these, the coupling protein VirD4 (TrwB) serves as the receptor to which both, DNA and protein substrates, bind before entry into the translocation channel (Cabezon and de la Cruz, 2006; Álvarez-Rodríguez et al., 2020; Costa et al., 2021). In conjugative systems, the substrate is a nucleoprotein formed by a protein called relaxase (TrwC in the case of R388 plasmid) and the conjugative DNA. This nucleoprotein substrate, together with some assistant proteins, form a complex known as the relaxosome (De La Cruz et al., 2010). Prior to translocation, the relaxase protein must be unfolded (Trokter and Waksman, 2018). The most structurally related protein to T3SS unfoldase InvC (SctN) is VirD4 (TrwB) (**Figure 4**). However, TrwB is a DNA-dependent ATPase (Tato et al., 2005) and, although it is directly involved in protein-DNA secretion (Llosa et al., 2002), an unfoldase role has never been assigned to this protein. In T4SS, VirB11 ATPase (TrwD) could participate in the unfolding of protein substrates (Cabezón et al., 2015). VirB11 belongs to the family of traffic ATPases (Planet et al., 2001) and shares a structural similarity to ATPases from other secretion systems, such as PilB or PilT from T4PS or GspE from T2SS (Peña and Arechaga, 2013).However, the mechanism and the forces that push and unfold substrates through the channel remain to be characterized. As in T3SS, an important aspect to be considered in powering the transport of the nucleoprotein complex is the contribution of the relaxase unfolding-refolding process. TrwC must be refolded in the recipient cell in order to re-circularize the ssDNA plasmid strand. Thus, a fast refolding of TrwC might also play an important role as a pulling force to complete the translocation process. Recent experiments, in which the co-translocational unfolding of a relaxase-DNA complex has been studied by nanopore technology, seem to point in that direction (Valenzuela-Gómez et al, 2023).

Work in T3 and T4SSs has uncovered a broad range of effector functions upon translocation (Pearson et al., 2015; Pinaud et al., 2018; Cover et al., 2020; Timilsina et al., 2020; Fromm and Dehio, 2021; Nandi et al., 2021). It is clear that in order to perform their function, effectors need to be active once in the host, meaning that protein refolding must occur after translocation. Thus, effectors have evolved folds that are compatible with basic requirements: they should be able to easily unfold, pass through the narrow secretion channel, and refold to an active form when on the other side (Metcalf et al., 2016; Gazi et al., 2021; LeBlanc et al., 2021). This is specially challenging in T3SS and T4SSs, since substrates are very large proteins. For instance, the relaxase of the F plasmid (TraI) has 1,756 amino acid residues and that of the R388 plasmid (TrwC), 966. The characterization of the mechanism of substrate transport is worthwhile due to its biological relevance, but there are still important open questions about how this process is occurring.

## A rotary catalytic mechanism for substrate secretion

Despite the differences in the structural architecture and in the type of substrates translocated by T3SS and T4SS, there is a significant structural homology between the main ATPase that powers substrate transfer in both secretion machineries. The cytosolic hexameric T3SS ATPase EscN from *E. coli* (InvC in *Salmonella* or SctN in the unified nomenclature) resembles the structure of the F_1_-ATPase (Zarivach et al., 2007; Majewski et al., 2019). Within the hexameric EscN ring there is a coiled-coil subunit named EscO (InvI/SpaM) that precludes the lumen of the ring. This arrangement is similar to that found in the F_1_-ATPase, where there is a γ subunit sitting in the center of the α/β heterohexamer (**Figure 4**). In the case of SctN, the ring is formed by a homohexamer, which also shares a high degree of structural similarity with the T4SS coupling protein TrwB (VirD4). The three proteins share a characteristic Rossmann fold, Walker A and B motifs and an hexameric stoichiometry (**Figure 4**). Thus, SctN and TrwB ATPases might be ancestral precursors to the heterohexameric rotary F_1_-ATPases, as previously proposed (Cabezon et al., 2012; Majewski et al., 2019).

EscN hexamer shows a marked asymmetry. The subunits present different occupancies in the active site and, subsequently, different conformational states. Thus, the six subunits would go through successive cycles of ATP binding, hydrolysis and ADP/phosphate release and the associated conformational changes at the C-terminal domain would be translated into torque on the EscO stalk (Majewski et al., 2019), in an analogous way to F_1_-ATPase. Since the interaction between the stalk and the export gate has been reported (Hara et al., 2012; Ibuki et al., 2013), it has been suggested that EscN might modulate the efficiency with which T3SS uses PMF. This finding also raises the possibility that elements of the export gate might function in a manner similar to the F_0_ components of the F_0_F_1_-ATPase, acting as a channel through which protons are pumped across the inner membrane (Majewski et al., 2019).

In the case of the T4SS ATPase homolog TrwB, six protein monomers associate to form an almost spherical quaternary structure that is also strikingly similar to F_1_-ATPase (**Figure 4**) (Gomis-Rüth et al., 2001), but with a six-fold symmetry. In F_1_- and EscN ATPases, protein structural asymmetry supports the rotary catalytic mechanism previously explained, but no structural asymmetry of the ring subunits has been found in TrwB to support a similar mechanism. However, it is tempting to propose that the asymmetry caused by the γ-subunit in F_1_-ATPase or the EscO subunit in EscN ATPase can also be induced in TrwB after DNA binding (**Figure 4**) (Cabezon and de la Cruz, 2006). Thus, such asymmetry would be observed in the active form of the protein, with a single DNA strand inside the central channel being translocated in a 5´- 3´direction. DNA might bind to a subunit in an “open” conformation (without ATP in the active site) and, following the binding-change mechanism, the subunit would change to a “closed” conformation. By doing so, the DNA would be sent into the internal cavity, being also rotated within the channel towards another catalytic subunit. Thus, a DNA pumping mechanism would take place iteratively, facilitating DNA transport through the T4SS. It is also important to note that DNA promotes TrwB oligomerization and activates its ATPase activity, in the same way as EscO enhances EscN oligomerization and ATPase activity in T3SSs. However, to date no experimental evidence is available to support this rotary mechanism for TrwB DNA pumping. Structural determination of TrwB in the presence of DNA could provide important insights in this regard.

## Important aspects in the search of common inhibitors for both secretion systems

Based on the structural similarity between T3SS and T4SS, it should be possible to find inhibitors that target different pathogens simultaneously. As only pathogenic bacteria express these secretion systems, non-pathogenic bacteria would not be targeted (Blasey et al., 2023). Salicylidene acylhydrazides (SAHs), for instance, are a group of chemical inhibitors active against the T3 and T4SSs of several pathogens (Mühlen and Dersch, 2020). Interestingly, the target of some SAH derivatives in T4SS is the inner membrane protein VirB8, which does not present structural similarity with any of the inner membrane components of T3SSs (**Figures 1**, **2**).

Other T3SS inhibitors such as thiazolidinone, which inhibits needle formation, also work on T2SSs. Since in these secretion systems the OM ring consists of pore-forming proteins that belong to the family of secretins, it is likely that this protein is the target of those compounds (Felise et al., 2008). A recent extensive review (Blasey et al., 2023) covers in depth the effects of many different inhibitors in both, T3 and T4SSs. Thus, it is not the aim of this work to focus on such analysis. While it is clear these chemical compounds inhibit effector secretion, more work is required to know the specific targets.

In that sense, ATPases are targets of great interest, since they are essential to power substrate translocation. *In silico* structure-based strategies to search for potential inhibitors from available libraries are a tool with great potential. Thus, it is possible to computationally screen millions of drug-like compounds and identify novel and high specific inhibitors. Ideally, inhibitors should operate through a non-competitive mechanism, in order to avoid off-target ATPases essential for cell metabolism. Small aromatic chemical compounds have been found to inhibit T3SS EscN ATPase and homologs from *Shigella* and *Salmonella (*Spa47 and FliI proteins, respectively), with an IC_50_ value of 25 µM (Case et al., 2020). A similar approach also allowed to identify two inhibitors of the homolog ATPase in *Chlamidya* (SctN), with IC_50_ values ~50-100 µM (Grishin et al., 2018) and in YscN ATPase from Yersinia, with IC_50_ values of ~20 µM (Swietnicki et al., 2011).

In T4SSs, most of the inhibitors against ATPases found so far have VirB11 ATPases as molecular target. Some examples are CHIR-1 compound (Hilleringmann et al., 2006) and also 8-amino imidazo[1,2-a]pyrazine derivatives (Sayer et al., 2014; Sayer et al., 2021), which act as inhibitors of the activity of HP0525 ATPase in *Helicobacter pylori*, with IC_50_ values in the rage 6 - 48 µM. Other examples are fatty acid derivatives, such as 2-hexadecanoic or 2-bromopalmitic acids, inhibitors of TrwD ATPase in R388 plasmid (IC_50_ values of ~20 µM) (Cabezón et al., 2017; García-Cazorla et al., 2018).

While micromolar IC_50_ values are not optimal for the development and use of these ATPase inhibitors as therapeutics, the identification of non-competitive inhibitors with low cellular toxicity is a major breakthrough, as these compounds would not affect the activity of F-type and other essential ATPases, compromising cell viability.

## Conclusion

The objective of this review is to highlight common aspects shared by T3 and T4SSs that might help in understanding their molecular mechanism and, most importantly, in the pursuit of inhibitors capable of targeting these systems. Certainly, there are notable distinctions between them. For instance, T4SSs lack a continuous channel in the periplasm, unlike T3SSs. Additionally, T3SSs do not support DNA transfer and, more importantly, there is no reported utilization of PMF as an energy source for substrate transfer in T4SSs. These are just a few of the numerous differences between the two systems, with several components exhibiting no homology. However, it is worth highlighting the homology observed in one of the primary ATPases that serves as a power supply in both systems. In that sense, it is important to note that in T3SSs, EscN ATPase is capable of energizing secretion even in the absence of bulk PMF (Terashima et al., 2018). In T3SSs, a rotary catalytic mechanism for substrate transfer has been proposed, based on the strong parallelism observed with the F_1_-ATPase (Majewski et al., 2019). As proposed here, a similar mechanism might also work for T4SSs.

Notable progress has been made in identifying inhibitors for individual secretion systems. However, it would be of great interest to discover inhibitors that can effectively target both secretion systems simultaneously. A promising starting point for this endeavor would be EscN/TrwB ATPases, found in T3 and T4SSs, respectively. Employing structure-based drug design methods will play a crucial role in developing more efficient compounds for this shared protein target, ideally achieving IC_50_ values in the nanomolar range to enhance their efficacy.

## Author contributions

EC: Conceptualization, Funding acquisition, Investigation, Project administration, Supervision, Writing – original draft, Writing – review & editing. FV: Investigation, Writing – original draft. IA: Investigation, Writing – original draft, Conceptualization, Funding acquisition, Supervision, Validation, Writing – review & editing.
